# Associations Between Active Myofascial Trigger Points, Electromyographic Activity and Kinesiophobia in Chronic Non-Specific Neck Pain

**DOI:** 10.3390/healthcare14101427

**Published:** 2026-05-21

**Authors:** Julián Müller-Thyssen-Uriarte, María Orosia Lucha-López, César Hidalgo-García, Rocío Sánchez-Rodríguez, Lucía Vicente-Pina, Loreto Ferrández-Laliena, Sofía Monti-Ballano, Pierre Vauchelles-Barré, José Miguel Tricás-Moreno

**Affiliations:** 1 Research Unit in Physiotherapy, Department of Physical Medicine and Nursing, University of Zaragoza, Domingo Miral, 50009 Zaragoza, Spain; jmuller@unizar.es (J.M.-T.-U.); r.sanchez@unizar.es (R.S.-R.); l.vicente@unizar.es (L.V.-P.); lferrandez@unizar.es (L.F.-L.); smonti@unizar.es (S.M.-B.); jmtricas@unizar.es (J.M.T.-M.); 2 Spin-off OMT-E Clinical Center Physiotherapy SLP, University of Zaragoza, Domingo Miral, 50009 Zaragoza, Spain; pierrevauchelles@gmail.com

**Keywords:** chronic non-specific neck pain, active myofascial trigger point, analgesic consumption, kinesiophobia, electromyographic activity

## Abstract

**Highlights:**

**What are the main findings?**

- The presence of active myofascial trigger points (A-MTrPs) could be associated with higher electromyography activity in sternocleidomastoid muscle of individuals with chronic non-specific neck pain (CNSNP) during craniocervical flexion test (CCFT).
- Analgesic consumption and kinesiophobia could be related to cervical muscle EMG activity during the CCFT and arm abduction test.

**What are the implications of the main findings?**

- A-MTrPs may contribute to neuromuscular alterations in cervical muscles and could be considered in the assessment and treatment of CNSNP.
- Psychosocial factors and demographic characteristics, such as kinesiophobia and analgesic consumption, may be included in the evaluation of motor control and designing rehabilitation strategies for individuals with CNSNP.

**Abstract:**

**Introduction**: Chronic non-specific neck pain (CNSNP) is a prevalent condition where active myofascial trigger points (A-MTrPs) are commonly detected in cervical muscles and may be associated with altered electromyographic activity (EMGact). However, their association with EMGact during functional tasks remains unclear. **Objectives**: This study aimed to explore this relationship, hypothesizing that A-MTrPs in cervical muscles would be associated with altered EMGact. **Methods**: An analytical cross-sectional exploratory study was conducted in 52 patients with CNSNP. Surface EMGact of the sternocleidomastoid (SCM), anterior scalene (AS), and upper trapezius (UT) muscles was recorded during the craniocervical flexion test (CCFT) and an isometric shoulder abduction task (ABD-90). Linear mixed-effects models were constructed to identify factors associated with EMGact. Age, pain intensity, pain duration, analgesic dose, anti-inflammatory dose, and kinesiophobia score were included as covariates, while gender, physical activity level, and the presence or absence of A-MTrPs were included as categorical factors. **Results**: At the 22 mmHg CCFT level, analgesic consumption was positively associated with peak EMGact and average AS activation (B = 0.791 and B = 0.223, respectively) and with SCM peak EMG act (B = 0.510). At the same level, kinesiophobia was associated with average SCM EMGact (B = 0.231). At the 26 mmHg CCFT level, average AS activation remained positively associated with analgesic consumption (B = 0.148) and SCM without A-MTrPs was associated with lower EMGact compared to SCM with A-MTrPs. At the 30 mmHg CCFT level, kinesiophobia was negatively associated with average EMGact of AS. In the UT muscle, during ABD-90, kinesiophobia was negatively associated with both peak (B = −0.378) and average EMGact (B = −0.132). **Conclusions**: The presence of A-MTrPs may be related to SCM EMGact during CCFT in individuals with CNSNP, while analgesic consumption and kinesiophobia also could be associated with cervical muscles EMGact during functional tasks.

## 1. Introduction

Neck pain (NP) may result from specific pathologies such as neurological, vascular, or inflammatory disorders, as well as fractures or intervertebral disc herniation. However, most cases lack an identifiable underlying cause and are thus classified as non-specific neck pain [1]. Based on symptom duration, chronic non-specific neck pain (CNSNP) is generally characterized by pain that persists or recurs for a period exceeding three months [2].

Globally, NP prevalence, including chronic and acute and specific and non-specific, remained relatively stable between 1990 and 2017, with an age-standardized point prevalence of 3551.1 per 100,000 population in 2017, corresponding to approximately 288.7 million cases worldwide [3].

In Spain, the prevalence of CNSNP—irrespective of underlying etiologies—decreased from 23.6% in 2006 to 12.3% in 2020, with persistently higher rates observed among women, older adults, and individuals of lower socioeconomic status [4].

Emerging evidence suggests that both psychosocial and biological factors contribute to the onset or persistence of NP. Psychological determinants include stress, maladaptive cognitive patterns, and sleep disturbances, whereas biological contributors encompass pre-existing neuromuscular or autoimmune conditions, aging, and genetic susceptibility [5].

A recent systematic review [6] identified clinical guidelines for neck and low back pain across several European countries. For neck pain, high-quality guidelines consistently recommend the use of oral analgesics and topical medications as part of evidence-based management strategies. These include commonly used painkillers such as paracetamol, non-steroidal anti-inflammatory drugs (recommended only for acute pain), and opioids (also restricted to acute pain conditions).

It has been suggested that the association between fear of movement and reduced upper trapezius (UT) electromyographic activity (EMGact) is stronger in patients with neck pain reporting higher pain levels, supporting the idea that decreased muscle activation may reflect an avoidance strategy to limit the use of painful muscles [7]. In addition, higher levels of kinesiophobia have been strongly associated with increased pain intensity, proprioceptive deficits, and reduced functional performance in individuals with CNSNP [8].

Myofascial trigger points (MTrPs) could be active or latent. An active myofascial trigger point (A-MTrP) is a trigger point that, upon stimulation, reproduces any symptom familiar to the subject, either partially or completely. In contrast, a latent MTrP does not reproduce any familiar symptoms when stimulated [9]. A-MTrPs are common findings in individuals with CNSNP: Chiarotto et al. [10] reported that pooled estimates of A-MTrP prevalence were highest in the UT, followed by the levator scapulae, sternocleidomastoid (SCM), and temporalis muscles. A systematic review by Lluch et al. [11] found that the prevalence of A-MTrPs ranged from 14% to 47% in the UT and from 0% to 65% in the levator scapulae in individuals with CNSNP. In other neck and shoulder muscles—including the splenius capitis, semispinalis capitis, scalene, multifidi, rhomboids, and SCM—the prevalence did not exceed 30%. Based on these findings, Lluch et al. concluded that A-MTrPs represent a prevalent and clinically relevant feature among individuals with NP.

Previous research has extensively described alterations in motor control patterns of the shoulder and neck muscles during various tasks in individuals with NP. During arm elevation tasks, individuals with NP exhibit increased EMGact of the UT [12,13,14] and lower trapezius [15] compared with healthy controls, also during cervical movement [16]. Prolonged cervical extensors activation [17,18] and delayed UT peak activity [19] have also been observed in those patients compared with asymptomatic people. However, in contrast, other studies have shown decreased activation of UT during bilateral reaches [20,21], decreased EMG amplitude in one side of the UT during repetitive upper limb tasks [13,22], lower middle trapezius activity [23] during overhead task, reduced EMGact in the semispinalis cervicis muscle during circular cervical contractions [24] and decreased EMGact of the scalene muscles [25] and cervical extensors [17] during cervical movements in patients with CNSNP.

Moreover, numerous studies have documented altered motor control patterns in cervical muscles among individuals with CNSNP during craniocervical flexion test (CCFT). In the posterior neck musculature, elevated EMGact of the UT [26] and splenius capitis [27] has been observed in CNSNP patients compared with healthy controls. In the anterior cervical region, increased EMGact of the SCM [26,27,28,29] and AS [27] muscles have also been consistently reported in CNSNP compared with asymptomatic subjects.

Previous research has suggested an association between the presence of latent MTrPs in the cervical musculature and altered motor function in healthy individuals. Ge et al. [30] reported that latent MTrPs in the UT were associated with accelerated muscle fatigue and increased loading of adjacent active motor units during sustained isometric shoulder abduction. In a subsequent study, Ge et al. [31] found that intramuscular, but not surface, EMGact in the UT was significantly greater at latent MTrP sites—both at rest and during shoulder abduction—compared with non-MTrP regions in healthy participants. These findings support the notion that latent MTrPs may contribute to subtle neuromuscular alterations even in the absence of clinical pain.

To date, and to the best of our knowledge, only one study has investigated whether the presence of A-MTrPs in a muscle is accompanied by EMG alterations in patients with CNSNP. Wytrążek et al. [32] reported lower EMG signal amplitudes during maximal voluntary contraction of the UT —indicative of muscle weakness—in subjects with A-MTrPs compared with healthy controls. Conversely, higher EMG amplitudes were observed during the resting state, suggesting abnormal muscle excitability associated with A-MTrP activity in those patients.

Although numerous studies have demonstrated altered EMGact in neck muscles during arm elevation tasks [12,15,17,19,20,23,33] and during the CCFT [26,27,34] in individuals with CNSNP compared with healthy controls, none have specifically examined the association of A-MTrPs in cervical muscles on EMGact during these tasks, comparing muscles with and without A-MTrPs exclusively in patients with CNSNP.

Moreover, given the difference between A-MTrPs and latent MTrPs in their role in generating the patient’s pain and considering the evidence of altered EMG patterns in CNSNP, muscles containing A-MTrPs are expected to exhibit alterations in EMGact. This highlights a relevant research gap concerning the potential association of A-MTrPs with EMGact in this population.

Therefore, the present study aimed to investigate the relationship between the presence or absence of A-MTrPs and EMGact in the cervical muscles of individuals with CNSNP during isometric abduction test and the CCFT. We hypothesized that the presence of A-MTrPs in the UT, SCM, and AS muscles would be associated with lower EMGact during these tasks compared with muscles without A-MTrPs in those patients.

## 2. Materials and Methods

### 2.1. Study Design and Sample Size Calculation

An exploratory analytical cross-sectional study was conducted.

The a priori sample size calculation was performed using GPower software, version 3.1 (https://www.psychologie.hhu.de/arbeitsgruppen/allgemeine-psychologie-und-arbeitspsychologie/gpower, accessed on 14 March 2025), Heinrich Heine Universität Düsseldorf (Düsseldorf, Germany), last accessed on 18 May 2026. The following analysis pathway was selected: test family, *t* tests; statistical test, Means: Difference between two independent means (two groups); type of power analysis, A priori: Compute required sample size—given α, power, and effect size. A two-tailed test was specified. The input parameters were effect size d = 2.00, α error probability = 0.05, statistical power = 0.95, and allocation ratio N2/N1 = 0.90. The effect size was calculated in GPower from the following values: mean group 1 = 27, mean group 2 = 14, and common within-group standard deviation = 6.5.

These mean difference and standard deviation values were based on the difference observed in average EMGact between the UT muscle with A-MTrPs and the UT without A-MTrPs reported by Wytrazek et al. [32]. The resulting required sample size was 8 participants per group, with a total sample size of 16 participants. A final sample of 52 patients was recruited to improve sample representativeness.

### 2.2. Participants

The sample comprised 52 patients with CNSNP (Table 1) recruited via informational banners at a local physiotherapy clinic and a primary care health center between March 2024 and July 2025.

Inclusion criteria were age ≥ 18 years and a history of NP lasting more than 3 months without a known pathological cause (e.g., traumatic, neurological).

Exclusion criteria included were history of major cervical trauma or recent surgery; pregnancy; generalized pain; ongoing litigation; diagnosed musculoskeletal, inflammatory, hormonal, or neurological disorders; severe psychiatric illness; inability to complete the questionnaire in Spanish; presence of a pacemaker; or physiotherapy treatment for their condition within the previous month.

The study protocol adhered to the principles of the Declaration of Helsinki and was approved by the Ethics Committee for Clinical Research of Aragon (CEICA), under protocol reference “Acta No. 13/2022,” with an approval date of 19 June 2022. All participants provided written informed consent prior to enrolment. Additionally, the study was registered and approved in ClinicalTrials.gov, with the following ID: NCT06257992.

### 2.3. Identification of Trigger Points

Two physiotherapists with over five years of experience in MTrP identification assessed the participants. Bilateral examination was performed on the sternocleidomastoid, anterior scalene, and upper trapezius muscles. Both examinators were blinded to EMG results.

The identification of MTrP locations in the upper trapezius has demonstrated moderate to good reliability, with intraclass correlation coefficients ranging from 0.62 to 0.81, indicating moderate to good agreement [35]. In addition, a study by Mayoral et al. [36] evaluated the interrater reliability of MTrP diagnosis in different muscles, including the sternocleidomastoid. The agreement between the two examiners was excellent for the SCM (K = 0.96).

The diagnostic criteria for MTrPs followed the Delphi consensus for international clinical standards [9] and included:

1. Presence of a palpable taut band;
2. Hypersensitive nodule within the taut band;
3. Referred pain.

The assessment procedure involved palpation to identify a taut band or hypersensitive nodule, followed by application of a digital algometer (Somedic AB Farsta, Somedic SenseLab AB, Sösdala, Sweden). Pressure was gradually increased perpendicular to the site at a rate of 1 kg/cm^2^/s until local and/or referred pain was elicited. If the elicited pain reproduced the participant’s neck symptoms (anterior or posterior), the MTrP was classified as active (A-MTrP), consistent with the methodology described by Simons [37].

### 2.4. Clinical Measures

Demographic data were collected through an online questionnaire, including age, gender, physical activity, analgesic and anti-inflammatory consumption as these variables are usually considered potential confounders in studies of CNSNP [38].

Participants were asked to indicate whether they had consumed any medication. If the response was affirmative, they were further asked to specify the type of medication taken (analgesics and/or anti-inflammatory drugs) and to report the number of pills consumed per month for each medication.

Physical activity levels were also assessed through the online questionnaire. Participants were asked to answer the following question: “How often do you engage in physical activity per week?” Response options corresponded to the frequency of weekly activity and included never, 1–2 times per week, 3–4 times per week, and 5 or more times per week.

Pain characteristics including intensity assessed with the Visual Analog Scale (VAS) and duration expressed in months to characterized chronicity were also recorded.

The VAS consists of a 10 cm line with endpoints representing “no pain” and “worst imaginable pain.” Patients mark the point that best reflects their perceived pain intensity, which is quantified by measuring the distance from “no pain” to the patient’s mark [39]. The VAS is a widely used generic measure of pain with good psychometric properties and is often considered the gold standard for pain assessment [40].

In addition, kinesiophobia was evaluated using the Spanish version of the Tampa Scale for Kinesiophobia (TSK-17). The scale has shown adequate internal consistency (Cronbach’s α = 0.79) in patients with chronic pain [41]. TSK-17’s score system ranges from 17 to 68 points; the higher the score, the higher the patient’s fear of movement/(re)injury. When the score is superior to 37 points, it is used as a reference threshold to indicate that the patient suffers from kinesiophobia [42].

### 2.5. Electromyography Acquisition

A surface electromyography system (Trigno Avanti, Delsys Europe, Manchester, UK) was used to record data on muscle activation patterns. Sensors were placed on the skin by a specific sticker after cleaning it with alcohol and cotton wool and waxing the area if necessary [43].

The examiners responsible for the assessment of MTrPs were blinded to the EMG results, as EMG data acquisition was conducted by a third examiner using the EMGworks Acquisition 4.8.0 software package (Delsys Europe, Manchester, UK).

Furthermore, the assessment of A-MTrPs was performed after completion of the functional tests used for EMG signal acquisition. Consequently, the examiner responsible for monitoring and recording the EMG signals was also blinded to the presence of A-MTrPs in the cervical musculature.

Surface electrodes were placed bilaterally in accordance with standardized placement guidelines. Electrodes for the SCM and AS muscles were positioned following the protocol described by Falla et al. [44], while electrode placement for the UT was conducted according to the SENIAM recommendations [45].

EMGworks Acquisition software 4.8.0 (Delsys Europe, Manchester, UK) was employed to collect the data. Before performing the tests, all sensors were calibrated to avoid recording noise and to obtain a relaxed muscle signal between −10 and +10 μV. EMG muscle activation data were exported to C3D format and processed using Visual3D software (HAS Motion, Kingston, ON, Canada).

### 2.6. Craniocervical Flexion Test

For CCFT, participants were positioned in the supine position with the knees flexed and the head and neck maintained in neutral alignment. They were first instructed to perform a slow and controlled craniocervical flexion movement, resembling a gentle nodding motion (“as if saying yes”), across three incremental stages of increasing pressure targets (22, 26, and 30 mmHg). These stages were adapted from the original five-stage protocol described by Jull et al. [46] to minimize muscular fatigability.

**Figure 1:**
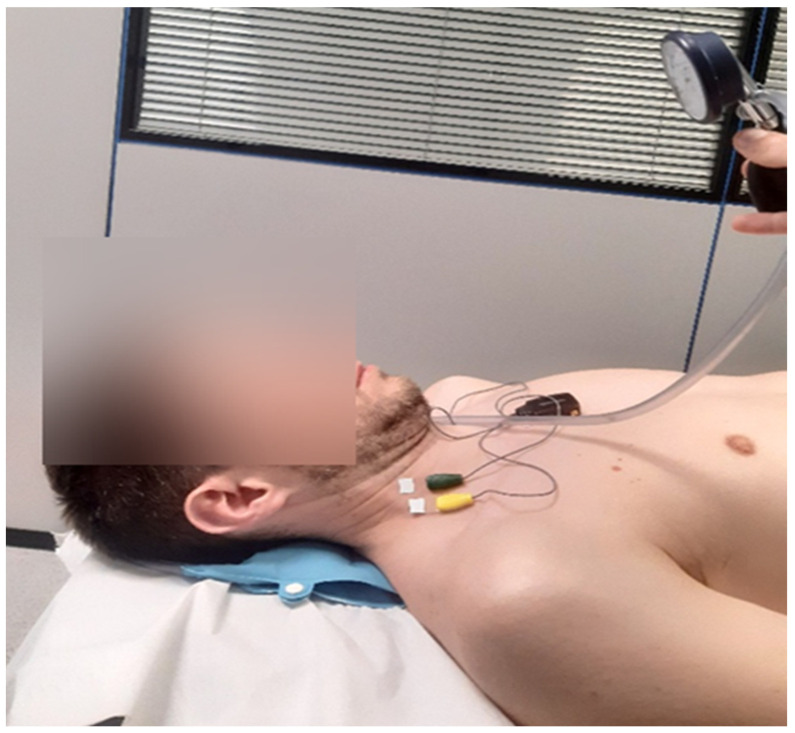
Electrodes were placed over the lower portion of the anterior scalene and sternocleidomastoid muscles for electromyographic recording, following the recommendations of Falla et al., (2002) [44] during the craniocervical flexion test.

Performance was guided by visual feedback provided through an air-filled pressure biofeedback unit (Stabilizer™, Chattanooga Group Inc., Hixson, TN, USA) positioned beneath the cervical spine and inflated to a stable baseline pressure of 20 mmHg (Figure 1). CCFT assesses both activation and endurance capacity of the deep cervical flexor muscles through progressive inner-range CCFT. Participants performed gentle nodding actions to gradually increase pressure to 22 mmHg, 26 mmHg, and finally 30 mmHg. At each target level, participants sustained a 10 s isometric contraction to assess muscular endurance [46] with a 20 s rest interval between stages. Increased activity in the superficial flexor muscles indicates a decrease in DCF activity during the CCFT [47].

During the isometric phase, surface electromyographic activity of the anterior scalene and sternocleidomastoid muscles was recorded. Consequently, the following EMG variables were extracted, all of which were expressed as a percentage of microvolts:

AS_22_PEAK, AS_26_PEAK, and AS_30_PEAK correspond to the peak activity of the anterior scalene at 22, 26, and 30 mmHg levels of the CCFT, respectively.

AS_22_AVG, AS_26_AVG, and AS_30_AVG represent the average activity of the anterior scalene at 22, 26, and 30 mmHg levels of the CCFT, respectively.

SCM_22_PEAK, SCM_26_PEAK, and SCM_30_PEAK correspond to the peak activity of the sternocleidomastoid at 22, 26, and 30 mmHg levels of the CCFT, respectively.

SCM_22_AVG, SCM_26_AVG, and SCM_30_AVG represent the average activity of the sternocleidomastoid at 22, 26, and 30 mmHg levels of the CCFT, respectively.

### 2.7. Shoulder Isometric Abduction Test at 90 Degrees

**Figure 2:**
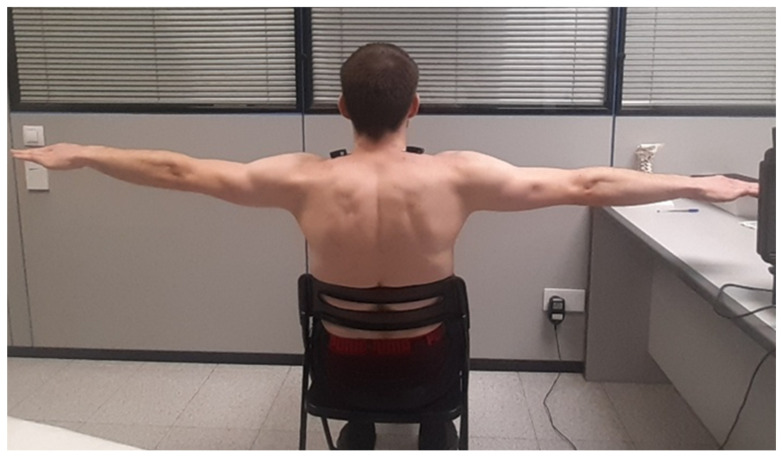
Electrode placement at 50% of the line between the acromion and the spinous process of C7, in accordance with SENIAM recommendations for electromyographic recording of the upper trapezius during the 90° shoulder isometric abduction test.

Upper trapezius EMGact was assessed during an isometric ABD-90 (Figure 2). Participants were seated with their backs fully supported, feet placed parallel on the floor, and hands resting on their knees. Upon verbal cue, they were instructed to abduct both arms to 90° of shoulder elevation with the palms facing downward and to maintain this position for 30 s. Consequently, the following EMG variables were extracted, all of which were expressed as a percentage of microvolts:

UT_ABD90_PEAK corresponds to the peak activity of upper trapezius during the shoulder isometric abduction test at 90 degrees.

UT_ABD90__AVG corresponds to the average activity of upper trapezius during the shoulder isometric abduction test at 90 degrees.

### 2.8. EMG Outcomes Variables and Data Analysis

Two variables were extracted from the EMG activity signal for muscle activation analysis: the peak EMGact and the average EMGact during the ABD-90 and CCFT tasks of each muscle tested.

EMGact of UT during the isometric ABD-90 and EMGact of SCM and AS during the CCFT was recorded and processed.

The sampling rate registration was 2000 Hz, and the common mode rejection ratio was 100 dB. The raw EMG signal was processed to enable the comparison of the statistical analysis. EMG signals were filtered using a second-order Butterworth high-pass filter with a cut-off frequency of 40 Hz to minimize movement artifacts and then full-wave rectified and low-pass filtered with a 15 Hz cut-off frequency [48,49].

The use of maximum EMG activation across trials was chosen as a normalization strategy due to the neck pain reported by participants, which may have limited the ability to reliably perform true maximal voluntary isometric contractions (MVIC). As MVIC-based normalization may be influenced by inter-individual variability in maximal effort and may not always reflect true maximal activation in clinical populations [50], we opted for a task-based alternative approach. Specifically, the maximum peak EMG amplitude obtained across all trials for each test (ABD-90 and CCFT), and for each side in each participant, was used as a reference value. This approach is consistent with previously described dynamic normalization techniques, which use task-derived EMG values when traditional MVIC procedures are not feasible or reliable [51].

For peak EMGact, the mean of the peak values derived from the envelope signal was first calculated across all trials for each test. This value was then normalized to the maximum EMG amplitude identified across all isometric phases from envelope signal and expressed as a percentage of this reference. Accordingly, peak EMG was reported as %max EMG for the UT during ABD-90, and for the SCM and AS during CCFT.

For average EMGact, the mean of the rectified EMG signal during the isometric phase of each trial was calculated and then averaged across trials. This value was then normalized to the maximum EMG amplitude identified across all isometric phases from the rectified signal and expressed as a percentage of this reference. Thus, average EMGact was also reported as a percentage of maximum EMG for UT during ABD-90 and for SCM and AS during CCFT.

The right and left sides of each muscle were analyzed separately, resulting in 104 measurements, corresponding to both sides of each muscle from the original 52 participants. In this context, muscles were classified into two groups, based on the presence or absence of active myofascial trigger points (with A-MTrPs or without A-MTrPs).

### 2.9. Statistical Analysis

Data were analyzed using IBM SPSS Statistics version 25 (IBM Corp., Armonk, NY, USA). Normality of the variables was assessed using the Shapiro–Wilk test. Descriptive statistics were calculated for all variables. Quantitative variables are presented as means and standard deviations (SDs), while qualitative variables are reported as frequencies and percentages.

To examine factors associated with EMGact of cervical muscles, a series of linear mixed models (LMMs) were constructed. Separate models were developed for the AS, SCM, and UT muscles, considering both peak and mean EMG values obtained during the corresponding tests as dependent variables.

Subject was included as a random effect, and the same covariates were initially entered into all models.

Age, pain intensity, pain duration, analgesic dose, anti-inflammatory dose, and kinesiophobia score were included as continuous covariates, while gender, physical activity level, and presence or absence of active myofascial trigger points (with A-MTrPs/without A-MTrPs) were included as categorical factors.

A second set of models was subsequently performed, including only the covariates that were statistically significant in the initial models, together with the main predictor variable (presence or absence of A-MTrPs).

Statistical significance was set at *p* < 0.05 with a 95% confidence level.

## 3. Results

### 3.1. Clinical and Demographic Characteristics of the Sample

Table 1 summarizes the demographic, clinical, and psychological characteristics of 52 participants with CNSNP. The mean age was 51.04 years; ages range widely, from a minimum of 21 to a maximum of 70, showing that the sample includes both relatively young adults and older individuals. Looking at percentiles, 25% of the sample is younger than 47.25 years, half are younger than 55, and 75% are younger than 59.5. This means the central 50% of ages lies between about 47 and 60 years. Women represented most of the sample (69.2%), compared with 30.8% men. Regarding physical activity, 28.8% of participants reported never exercising, 23.1% exercised 1–2 times per week, 34.6% exercised 3–4 times per week, and 13.5% exercised five or more times per week. Participants reported a mean analgesic consumption of 2.58 pills per month and a mean anti-inflammatory consumption of 7.75 pills per month. The average NP intensity was 4.75, indicating moderate pain, and the mean duration of symptoms was 103.29 months, reflecting long-standing chronic pain. The distribution of A-MTrPs varied between muscles. In the AS, most were without A-MTrPs (80; 76.9%), while those with A-MTrPs accounted for 24 cases (23.1%). In the SCM, the distribution was more balanced, with 49 muscles without A-MTrPs (47.1%) and 55 with A-MTrPs (52.9%). In contrast, UT showed a predominance of A-MTrPs (88; 84.6%), with only 16 muscles without A-MTrPs (15.4%). Finally, kinesiophobia (TSK-17) showed a mean score of 35.79, suggesting a moderate-to-high level of fear of movement.

All 52 participants were included in the analyses; however, some missing data were observed in the EMG variables. To determine the extent of this missingness, a univariate analysis was conducted for all EMG variables.

The univariate analysis revealed varying proportions of missing data across the studied variables. Among the peak measurements, AS_22_PEAK, AS_30_PEAK, and SCM_22_PEAK each presented 6.7% missing values, while SCM_30_PEAK showed the highest proportion of missing data at 11.5%. In contrast, AS_26_PEAK and SCM_26_PEAK exhibited relatively low levels of missingness, with 2.9% and 3.8%, respectively. The variable UT_ABD90_PEAK had 7.7% missing data. Regarding the average measurements, SCM_30_AVG also displayed a high percentage of missing values (11.5%), followed by SCM_22_AVG (9.6%) and AS_22_AVG (8.7%). Moderate levels of missing data were observed in AS_FX30_AVG (6.7%) and UT_ABD90_AVG (3.8%), whereas AS_26_AVG and SCM_26_AVG showed lower proportions of missingness, with 3.8% and 1.9%, respectively.

The missing data were mainly due to failures in the data acquisition process through the EMG sensors, primarily caused by signal noise or by the patient’s inability to reach certain force levels during the test due to pain.

### 3.2. Univariable Linear Mixed Model (LMM) Analysis of Peak and Average EMG Activity in UT, SCM, and AS

For the AS muscle (Table 2), monthly analgesic consumption was the most consistent positive predictor of EMGact across tasks. At the 22 mmHg level, peak AS activation increased by 0.791 percentage points per additional pill the patient took, while average AS activation increased by 0.223 percentage points. At the 26 mmHg level, analgesic consumption remained positively associated with average AS activation (B = 0.148). No significant predictors were observed at 30 mmHg, except for kinesiophobia, which is negatively associated with AS mean activation; for each additional point that patient scored at TSK-17, the AS mean activation decreases 0.216 percentage points.

For the SCM muscle (Table 3), monthly analgesic consumption was a consistent positive predictor of EMGact at lower contraction levels. At the 22 mmHg level, peak SCM EMGact increased by 0.510 percentage points of activation per additional pill taken by the patient, and mean activation is associated with an increased by 0.177 percentage points.

At the 26 mmHg level, peak SCM activation was negatively associated with absence of A-MTrPs (B = −7.597); no consistent predictors were observed for average SCM activation at 26 mmHg.

At the 30 mmHg level, mean SCM activation was lower in patients who did not engage in exercise (B = −5.339) and in those who exercised 3–4 times per week (B = −4.474), compared with those who exercised more than five times per week. No consistent predictors were identified for peak SCM activation at 30 mmHg.

For the UT muscle (Table 4), kinesiophobia was negatively associated with peak activation (B = −0.378), indicating that each additional point on the TSK-17 is related to a decrease of 0.378 percentage points in UT peak activation.

For average UT activation, kinesiophobia was also negatively associated (B = −0.378), indicating that each additional point in TSK-17 score was associated with a reduction of 0.134 percentage points. Furthermore, gender was associated with UT average activation, with males showing 2.112 higher activation compared to females (*B* = 2.112).

## 4. Discussion

The aim of this study was to determine whether the presence of A-MTrPs is related to the EMGact of the UT, SCM, and AS muscles during two functional tasks in individuals with CNSNP.

Our initial hypothesis was partially supported. Specifically, in the SCM, the absence of A-MTrPs was associated with lower, rather than higher—as initially hypothesized—average peak EMGact compared with muscles presenting A-MTrPs at the 26 mmHg level of the CCFT. These findings contrast with those reported by Florencio et al. [52], who observed lower activation in superficial neck muscles (SCM and UT) in women with migraine presenting A-MTrPs during the CCFT. Nevertheless, our results are in line with previous studies reporting higher EMGact of the superficial cervical flexor muscles in individuals with CNSNP during the CCFT [26,27,34].

One potential mechanism underlying the association between pain intensity and increased EMG amplitude may involve heightened sympathetic activity at both latent and A-MTrPs [53], which could increase motor unit activity. Moreover, the prevalence of endplate noise has been shown to be higher in A-MTrPs compared with latent MTrPs, which may further reflect increased neuromuscular excitability [54]. It has been proposed that when a muscle contains latent MTrPs, motor unit hyperexcitability may contribute to the sustained activation of taut muscles bands, potentially promoting further dysfunction and accelerated fatigability during muscle contraction [55]. In this context, motor units within muscles with latent MTrPs may need to generate greater activity to achieve comparable force, possibly contributing to less efficient and more heterogeneous activation patterns among synergistic muscles [55].

In the present study, pain intensity is associated with EMGact during the CCFT. However, the relationship between cervical pain intensity and cervical muscle EMGact during the CCFT has been previously investigated. For example, Bonilla et al. [27] reported that greater pain-related disability was associated with increased EMGact of the AS and UT muscles during the CCFT in women with CNSNP. Similarly, two studies [33,56] have found a positive relationship between pain intensity and the activation of the SCM and AS during the CCFT, particularly at the final increments of the test. Falla et al. [33] also reported that higher pain levels were associated with lower activation of the deep cervical flexor muscles during the CCFT. Within this context, the present finding that the presence of A-MTrPs in the SCM may be associated with higher EMGact appears to be broadly consistent with previous observations. However, this interpretation should be made with caution, as differences in methodology and population characteristics—particularly the fact that no healthy subjects were included in the present study—may limit direct comparability.

Another relevant finding of this study is that analgesic consumption is a positively associated with peak and average EMGact of the AS muscle at the 22 and 26 mmHg pressure levels of the CCFT. In addition, analgesic consumption is significantly related to SCM peak and average EMGact at 22 mmHg during the CCFT.

There is currently limited evidence regarding the relationship between analgesic consumption and EMGact. However, Gruss et al. [57] investigated the effect of analgesic administration on facial muscle EMGact and other physiological signals in post-operative patients. Measurements were obtained five minutes after drug administration to investigate measurable physiological responses to pain relief. The authors reported that facial EMGact showed the strongest effect among all physiological responses, with a significant decrease in muscle activity following analgesic consumption. Important methodological differences should be considered when comparing those findings with the present study. In our study, analgesic consumption was quantified as the number of doses taken during the month prior to the assessment, whereas Gruss et al. [57] evaluated the short-term physiological effect immediately after drug administration. In contrast to their results, our findings indicate that higher analgesic consumption is associated with increased EMGact.

A possible hypothesis is that individuals who report higher consumption of analgesics per month may also experience greater levels of neck pain, thereby requiring more frequent pharmacological management. In this context, it could be speculated that during tasks such as the CCFT and the ABD-90 test, these individuals may experience increased pain during muscle contraction. Pain during muscle activation might reduce muscle efficiency, potentially leading to increased EMGact to maintain a given level of force output.

However, this interpretation should be considered with caution, as pain was not directly measured during task performance and the cross-sectional design of the study precludes any causal inference [58].

Chronic pain has been shown to induce escape and avoidance behaviors and is strongly associated with kinesiophobia [59]. However, the association of kinesiophobia with cervical muscle activation has been scarcely investigated. In the present study, higher levels of kinesiophobia is associated with reduced muscle activation. This relationship was observed for the average EMGact of the AS muscle at the 30 mmHg level of the CCFT, the average EMGact of the SCM muscle at the 22 mmHg level, as well as for peak and average EMGact of the UT during the ABD-90 test. Similar findings were reported by Nederhand et al. [60], who studied patients with acute traumatic neck injury following a motor vehicle accident. In their study, 92 patients performed 90° isometric arm abduction task, and UT EMGact was recorded at five time points (1, 4, 8, 12, and 24 weeks after the accident). The authors found that higher levels of pain and kinesiophobia were associated with lower EMGact. These findings are consistent with both the pain adaptation model [61] and the fear-avoidance model [62], which suggest that reduced muscle activation may represent a protective strategy aimed at avoiding the use of painful muscles and preventing further injury. Additional evidence supporting the relevance of kinesiophobia in CNSNP was reported by Asiri et al. [8], who found in a sample of 64 individuals with CNSNP that kinesiophobia predicted NP intensity, proprioception deficits, and functional performance measured through handgrip strength.

Gender also appeared to be related to muscle activation patterns. In our study, males showed significantly higher peak activation of the AS muscle at the 22 mmHg level of the CCFT, with values approximately 2.1 points in percentage of activation higher than those observed in females. These findings contrast with previous research. For example, Chen et al. [63] reported that women exhibited higher EMGact of cervical extensor muscles and the UT while using smartphones in different postures compared with men. Additionally, Nie et al. [64] reported greater fatigue resistance of the UT during high-intensity eccentric shoulder exercise in women. It is also important to contextualize these findings within epidemiological data indicating that the prevalence of NP is higher in women than in men worldwide [3], including in Spanish population-based studies [38]. Lower pressure pain thresholds in MTrPs of cervical and cranial muscles—potentially contributing to headache symptoms—have also been observed in women compared with men with tension-type headache. Moreover, mean PPT values tend to be lower in women than in men, suggesting sex-related differences in pain sensitivity and central pain processing [65].

Age also appears to be related with average EMGact of the AS muscle, reaching statistical significance during the CCFT at the 22 mmHg pressure level. Although few studies have directly examined the relationship between age and cervical muscle EMGact, age-related changes in muscle properties have been reported. For example, Kocur et al. [66] found that increasing age was associated with greater stiffness and tone and reduced elasticity of the UT and SCM muscles in women. In contrast, Cannon et al. [67], studying knee extensor EMGact in young and elderly women following a 10-week training program, did not observe significant differences between age groups. Finally, epidemiological evidence suggests that age is also associated with the occurrence of CNSNP. In a Spanish population-based study [38], individuals older than 35 years showed a higher prevalence of CNSNP, highlighting age as a possible relevant factor in the clinical profile of this condition.

The demographic characteristics of the sample showed that women comprised nearly 70% of participants, reflecting the typical gender distribution observed in CNSNP populations [38]. The mean age of participants was 51 years, which is close to the age group with the highest number of CNSNP cases reported in the Global Burden of Disease Study, 45–49 years [3].

Reduced physical activity is associated with an increase in the risk of cervical pain [5]. In our study, 28.8% of participants reported never engaging in physical activity, 23.1% exercised 1–2 times per week, 34.6% exercised 3–4 times per week, and 13.5% exercised five or more times per week.

The mean NP intensity measured using NPRS was 4.75, indicating a moderate level of pain, consistent with values typically reported in CNSNP populations [56]. The mean duration of NP was 103.29 months (SD = 118.27; 95% CI: 80.29–126.29), approximately 8.6 years, confirming that participants were experiencing long-standing chronic symptoms. Participants reported a mean analgesic intake of 2.58 pills per month and a mean anti-inflammatory intake of 7.75 pills per month. In population surveys from Spain, the use of analgesics and anti-inflammatory medications is significantly more prevalent among individuals with CNSNP (≈42% vs. ≈21% among those without pain), and pain in this region is significantly associated with such pharmacological use [68].

In our sample, A-MTrPs were most prevalent in the UT (84.6%), followed by the SCM (52.9%) and AS (23.1%). These findings are consistent with previous research demonstrating a high prevalence of A-MTrPs in the UT in CNSNP with approximately 82% presenting UT A-MTrPs [69].

Kinesiophobia, assessed using the TSK-17, showed a mean score of 35.79, very close to the scores above 37 that are generally considered to indicate kinesiophobia [42].

Several limitations should be acknowledged. First, the cross-sectional design prevents the establishment of causal relationships, and the study is largely exploratory and may be underpowered to detect certain associations observed in the results. Second, EMG measurements focused only on average and peak activation, while other relevant parameters, such as the onset of neck–shoulder muscle fatigue, were not considered. Third, potential differences in resting muscle activity or during other functional tasks (e.g., computer typing) between muscles with and without A-MTrPs were not assessed. Fourth, we should acknowledge as a limitation the inherent subjectivity of MTrP palpation, which may introduce some error in inter-examiner reliability; however, reliability testing was not formally conducted in the present study. Fifth, we observed that for some variables—such as gender at the 22 mmHg level of the CCFT for peak EMGact of the AS, analgesic consumption at the 26 mmHg level of the CCFT for average EMGact of the AS, and kinesiophobia at the 30 mmHg level of the CCFT for average EMGact of the AS—the low achieved statistical power may have limited the detection of additional statistically significant associations. The same applies to variables such as age and SCM without A-MTrPs at the 30 mmHg level of the CCFT for average EMGact, as well as during the ABD90 test for pain and UT without A-MTrPs for peak and average activity, respectively. Additionally, the use of a validated instrument such as the IPAQ questionnaire, rather than a single ordinal question, would have provided a more accurate assessment of physical activity levels in participants. Similarly, the method used to quantify analgesic consumption (i.e., number of pills per month) has important limitations, as it does not account for dosage, medication type, or pharmacological classification. Finally, the small sample size and the use of a convenience sample, together with absence of a healthy control group, limits our ability to determine whether the observed EMG patterns are specific to CNSNP or reflect normal physiological variation.

In this study, significant B coefficients ranged in magnitude, with some indicating relatively larger changes in muscle activation. However, there is currently no established evidence defining minimally clinically important differences for EMG-derived activation values in this context, and direct comparisons with previous studies are limited due to the novelty of the research area. Consequently, it remains difficult to determine whether the observed differences represent clinically meaningful functional effects in clinical practice.

Moreover, regarding EMG data analysis, it should be acknowledged that the task-specific normalization approach used in this study may limit comparability with other tasks or studies that use MVIC-based normalization in previously referenced studies. For instance, MVIC normalization has been applied in patients with CNSNP during the CCFT [27], as well as during arm elevation tasks [19,20,23]; therefore, comparability with those studies may be limited.

An additional consideration is the potential relationship of unmeasured confounders not included in the present model. In particular, body mass index may affect both muscle activation patterns and mechanical load during cervical tasks. Similarly, occupational factors (e.g., repetitive upper-limb tasks) may contribute to habitual loading of the cervical–shoulder region. Relatedly, ergonomic exposure—such as sustained forward head posture and workstation setup—has been associated with altered neck muscle activity and may modulate both pain and motor control strategies. The absence of these variables may limit the ability to fully attribute observed differences in EMGact exclusively to the clinical or experimental predictors included in the model. Future studies should incorporate these factors to better isolate the specific contribution of myofascial trigger points and psychological variables to neuromuscular function in CNSNP.

The clinical implications of this study should be interpreted with caution. Demographic characteristics—such as age, sex, analgesic consumption, and physical activity levels—may be considered when exploring factors potentially associated with the clinical profile of patients with CNSNP; however, their clinical relevance remains to be further established.

While a previous study [52] suggests that A-MTrPs may be associated with altered motor patterns, the potential relevance of addressing these points before other interventions, such as manual therapy of the cervical spine and/or exercise-based approaches targeting the activation of the deep cervical flexor muscles, should be interpreted cautiously, as a hypothesis that requires confirmation in future interventional studies. Finally, these findings may suggest the potential relevance of fear of movement in this population. Accordingly, interventions aimed at reducing kinesiophobia, such as graded exposure to movement and activity, could be considered as part of a broader therapeutic approach. Nevertheless, their specific effects on sensorimotor function and clinical outcomes remain uncertain and should be further explored in future research.

## 5. Conclusions

The results of this study suggest that the presence of A-MTrPs may be associated with differences in cervical muscle activation during functional tasks in individuals with CNSNP. Specifically, the presence of A-MTrPs in the SCM was related to higher EMGact during the CCFT.

Analgesic consumption and kinesiophobia also emerged as variables that could be associated with EMGact. Higher analgesic consumption was related to increased activation of the AS and SCM during the CCFT, whereas higher levels of kinesiophobia were associated with lower EMGact of the AS, SCM and UT during functional tasks. These associations should be interpreted as exploratory and do not imply causal relationships, warranting further investigation in future interventional studies.

## Figures and Tables

**Table 1:** Clinical and demographic characteristics of the sample.

Chronic Non-Specific Neck Pain *N* = 52		
Demographical Variables	Mean (SD)/n (%)	CI 95%
Age (years)	51.04 (11.87)	48.73 to 53.35
Gender, n (%)	Male, 16 (30.8%)	Female, 36 (69.2%)
Physical activity (times/week). *n* (%)	never. 15 (28.8%) 1–2 times/week. 12 (23.1%) 3–4 times/week. 18 (34.6%) 5 times or more/week. 7 (13.5%)	
Analgesic consumption (pills/months)	2.58 (8.57)	0.91 to 4.24
Anti-inflammatory consumption (pills/months)	7.75 (11.68)	5.48 to 10.02
Clinical variables		
Neck pain intensity (VAS scale 0–10)	4.75 (1.28)	4.50 to 5.00
Duration of neck pain (months)	103.29 (118.267)	80.29 to 126.29
AS n (%)	80 without A-MTrPs (76.9%)	24 with A-MTrPs (23.1%)
SCM n (%)	49 without A-MTrPs (47.1%)	55 with A-MTrPs (52.9%)
UT n (%)	16 without A-MTrPs (15.4%)	88 with A-MTrPs (84.6%)
Psychological variable		
Kinesiophobia (TSK-17 score)	35.79 (5.95)	34.63 to 36.94

SD: standard deviation; CI: confidence interval; VAS: Visual Analog Scale; AS: anterior scalene; SCM: sternocleidomastoid; UT: upper trapezius; A-MTrP: active myofascial trigger point; TSK-17: Tampa Scale for Kinesiophobia.

**Table 2:** Univariable LMM analyzing AS EMGact significant predictors during craniocervical flexion test (CCFT).

Univariable Analysis. Dependent Variable:	Significant Predictor	B	*p*
AS_22_PEAK	Analgesic consumption	0.791	0.005
AS_26_PEAK	No significant predictor		
AS_30_PEAK	No significant predictor		
AS_22_AVG	Analgesic consumption	0.223	0.000
	Age	−0.089	0.010
AS_26_AVG	Analgesic consumption	0.148	0.018
AS_30_AVG	Kinesiophobia	−0.216	0.011

AS_22_PEAK: peak activation of anterior scalene during 22 mmHg level of CCFT; AS_26_PEAK: peak activation of anterior scalene during 26 mmHg level of CCFT; AS_30_PEAK: peak activation of anterior scalene during 30 mmHg level of CCFT. AS_22_AVG: average activation of anterior scalene during 22 mmHg level of CCFT; AS_26_AVG: average activation of anterior scalene during 26 mmHg level of CCFT; AS_30_AVG: average activation of anterior scalene during 30 mmHg level of CCFT. B: beta coefficient.

**Table 3:** Univariable LMM analyzing SCM EMGact significant predictors during craniocervical flexion test (CCFT).

Univariable Analysis. Dependent Variable:	Significant Predictor	B	*p*
SCM_22_PEAK	Analgesic consumption	0.510	0.043
SCM_26_PEAK	SCM without A-MTrPs	−7.597	0.036
	SCM with A-MTrPs	Reference category	
SCM_30_PEAK	No significant predictor		
SCM_22_AVG	Analgesic consumption	0.177	0.012
	Kinesiophobia	0.231	0.023
SCM_26_AVG	No significant predictors		
SCM_30_AVG	Physical activity (never)	−5.339	0.004
	Physical activity (3–4 times/week)	−4.474	0.013
	Physical activity (≥5 times/week)	Reference category	

SCM_22_PEAK: peak activation of sternocleidomastoid during 22 mmHg level of CCFT; SCM_26_PEAK: peak activation of sternocleidomastoid during 26 mmHg level of CCFT; SCM_30_PEAK: peak activation of sternocleidomastoid during 30 mmHg level of CCFT; SCM_22_AVG: average activation of sternocleidomastoid during 22 mmHg level of CCFT; SCM_26_AVG: average activation of sternocleidomastoid during 26 mmHg level of CCFT; SCM_30_AVG: average activation of sternocleidomastoid during 30 mmHg level of CCFT. A-MTrPs: Active myofascial trigger points. B: beta coefficient.

**Table 4:** Univariable LMM analyzing UT EMGact significant predictors during ABD-90.

Univariable Analysis. Dependent Variable:	Significant Predictor	B	*p*
UT_ABD90_PEAK	Kinesiophobia	−0.378	0.024
UT_ABD90_AVG	Gender (male)	2.112	0.018
	Gender (female)	Reference category	
	Kinesiophobia	−0.134	0.047

UT_ABD90_PEAK: upper trapezius peak activity during isometric shoulder abduction at 90 degrees. UT_ABD90_AVG: upper trapezius average activity during isometric shoulder abduction at 90 degrees. A-MTrPs: active myofascial trigger point. B: beta coefficient.

## Data Availability

The data presented in this study are available on request from the corresponding author due to ethical reasons.

## Abbreviations

The following abbreviations are used in this manuscript:

NP	Neck pain
CNSNP	Chronic non-specific neck pain
MTrPs	Myofascial trigger points
UT	Upper trapezius
SCM	Sternocleidomastoid
A-MTrPs	Active myofascial trigger points
CCFT	Craniocervical flexion test
DCF	Deep cervical flexor muscle
EMG	Electromyography
AS	Anterior scalene
VAS	Visual Analog Scale
TSK-17	Tampa Scale for Kinesiophobia
EMGact	Electromyographic activity
ABD-90	Isometric shoulder abduction task at 90 degrees
LMM	Linear mixed model
MVIC	Maximal voluntary isometric contractions
